# The extracellular death factor (EDF) protects *Escherichia coli* by scavenging hydroxyl radicals induced by bactericidal antibiotics

**DOI:** 10.1186/s40064-015-0968-9

**Published:** 2015-04-16

**Authors:** Zhongyi Yan, Guodong Li, Yanfeng Gao, Wenjie Zhai, Yuanming Qi, Mingxia Zhai

**Affiliations:** School of Life Sciences, Zhengzhou University, 100 Science Road, Zhengzhou, 450001 China

**Keywords:** Extracellular death factor, Antioxidant, Reactive oxygen species, Antibiotics, Hydroxyl radicals, Peptide

## Abstract

The newly discovered extracellular death factor (EDF) is a pentapeptide with the sequence NNWNN in *Escherichia coli*. It was reported that it participated in the cell death process mediated by toxin-antitoxin system *mazEF*. Reactive oxygen species (ROS) are recently considered as common factors for bactericidal antibiotics-mediated cell death. Previous study indicated that EDF could scavenge hydroxyl radicals and might act as a signal molecule with dual effects, “death” and “survival”. But the structure-activity relationship of EDF and the effects of EDF on the activity of antibiotics remain unclear. In the present study, our results indicated that tryptophan could be the key residue to the hydroxyl radicals-scavenging activity of EDF, and EDF could protect *Escherichia coli* from killing by bactericidal antibiotics, but not by DNA-damaging or bacteriostatic antibiotics. Our results could provide novel evidence to understand the role of EDF in drug-resistance.

## Background

The *E*xtracellular *D*eath *F*actor (EDF) with the sequence NNWNN (Asn-Asn-Trp-Asn-Asn-OH) was discovered by Kolodkin-Gal and colleagues in 2007 (Kolodkin-Gal et al. 2007). EDF is the first peptidic molecule involved in the quorum-sensing of *E. coli*. It is different from the classical peptidic quorum-sensing molecules among gram-positive bacteria because that it is derived from an enzyme, glucose-6-phosphate dehydrogenase (Kolodkin-Gal et al. 2007; Kolodkin-Gal and Engelberg-Kulka 2008). EDF and the classical toxin-antitoxin system *mazEF* could determine the programmed cell death (PCD) mode of some bacteria induced by antibiotics (Kolodkin-Gal et al. 2008). It was reported that EDF could inhibit the formation of the MazEF complex, and thus enhance the endoribonucleolytic activity of MazF (Belitsky et al. 2011). The toxin MazF could lead to the PCD of the major population of *E. coli* (Amitai et al. 2009).

Kohanski et al. proposed a common mechanism that ROS (especially hydroxyl radicals) could be considerable factors on bacterial PCD which was triggered by bactericidal antibiotics, but not by bacteriostatic antibiotics (Kohanski et al. 2007). Our previous results showed that EDF could act as an antioxidant to scavenge hydroxyl radicals *in vitro* (Gao et al. 2010). Therefore, it is very necessary to study the effects of EDF on *E. coli* treated by antibiotics, and to investigate the structure-activity relationship and reaction rates of EDF to scavenge hydroxyl radicals.

## Methods

### Bacterial strain and peptides

Wild type *E. coli* MC4100 strain was obtained from China General Microbiological Culture Collection Center (CGMCC) (Number: 1.156). EDF and its glycine substituted mutants were synthesized by using a standard solid phase Fmoc-^t^Bu peptide synthesis strategy in our laboratory, and were purified to more than 95% purity by reverse phase high performance liquid chromatography. Their molecular weights were confirmed by electrospray ionization-mass spectrometry.

### Hydroxyl radicals-scavenging activity and the reaction rates of EDF and its mutants

2-deoxyribose can be oxidized by hydroxyl radicals triggered by Fenton reagents, and then the oxidized products of 2-deoxyribose can react with 2-thiobarbituric acid under heating condition to produce a pink chromogen (thiobarbituric acid reactive species, TBARS). The absorbance of TBARS can be detected at 532 nm. The assay was performed according to the method described previously (Mahakunakorn et al. 2004). The reaction mixture contained 20 mM KH_2_PO_4_–KOH buffer (pH 7.4), 2.8 mM 2-deoxyribose, 1.0 mM H_2_O_2_, and 100 μM FeCl_3_ premixed with 100 μM EDTA. The reaction was triggered by the addition of 100 mM ascorbic acid. The test compounds are, EDF and its mutants (0.01 mM, 0.03 mM, 0.1 mM, 0.3 mM, and 0.6 mM), thiourea (Sigma-Aldrich) (0.2 mM, 0.4 mM, 0.8 mM and 1.6 mM), and 2, 2′-dipyridyl (Sigma-Aldrich) (0.5 mM, 1 mM, 2 mM, 4 mM). After incubation for 60 min at 37°C, the absorbance was measured at 532 nm. The hydroxyl radicals-scavenging activity of the compound was represented as the inhibition percentage of 2-deoxyribose degradation. To calculate the reaction rates of the compounds, the previously reported equation was used (Halliwell et al. 1987; Cheng et al. 2003). Thiourea, 2, 2′-dipyridyl, and glucose were used as positive controls (Gutteridge et al. 1990).

### The bacterial viability assay treated by different kinds of antibiotics

The viability assay was performed according to the procedure described previously with minor modification (Kohanski et al. 2007). *E. coli* MC4100 cells were grown in Luria-Bertani (LB) medium at 37°C and 220 rpm in a light insulated shaker. When the value of optical density (OD_600_) reached 0.1, cells were diluted to 2 × 10^5^ cells/mL to avoid the generation of endogenous EDF. Then, EDF (0.1 μg/mL), each mutant of EDF (0.1 μg/mL), thiourea (150 mM), or 2, 2′-dipyridyl (0.5 mM), was added and incubated with each antibiotic (15 μg/mL ampicillin for 4 hours, 1 mg/mL nalidixic acid for 3 hours, or 40 μg/mL rifampicin for 4 hours). 800 μL of culture medium was collected, washed twice with PBS (pH 7.2), and then serially diluted in PBS. After incubation in LB medium at 37°C overnight, dilutions with 20–80 colonies/well were counted. The CFU/mL values were calculated.

### Effects of EDF on the hydroxyl radicals produced in *E. coli*

The production of hydroxyl radicals in *E. coli* was detected by using a flow cytometer (FACSCalibur, Becton Dickson). 3′-(*p*-hydroxyphenyl) fluorescein (HPF, Sigma-Aldrich, 5 μg/mL) was used as probe to detect the hydroxyl radicals, and it was added as along with the antibiotics. Thiourea and 2, 2′-dipyridyl were used as positive controls.

### Statistical analysis

All data were presented as means ± S. D. (n ≥ 3). The statistical significance of difference between each group was analyzed by Student’s *t* test. The statistical significances were presented as *p < 0.05, **p < 0.01, and ***p < 0.001, respectively.

## Results

### Hydroxyl radical-scavenging activity of EDF and its mutants

**Figure 1:**
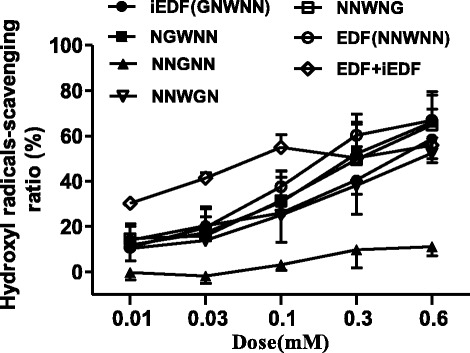
The hydroxyl radicals-scavenging activity of EDF and its mutants substituted by glycine. iEDF is the inhibitor of EDF to its quorum-sensing effects. Data were presented as means ± S.D. (*n* = 3).

**Figure 2:**
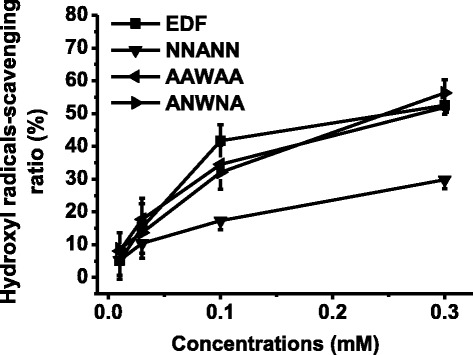
Hydroxyl radicals-scavenging activity of EDF (NNWNN) and its alanine substituted mutants. The hydroxyl radicals scavenging activity values represented the percentage of inhibition of deoxyribose degradation. Data were expressed as means ± S.D. (*n* = 3).

**Figure 3:**
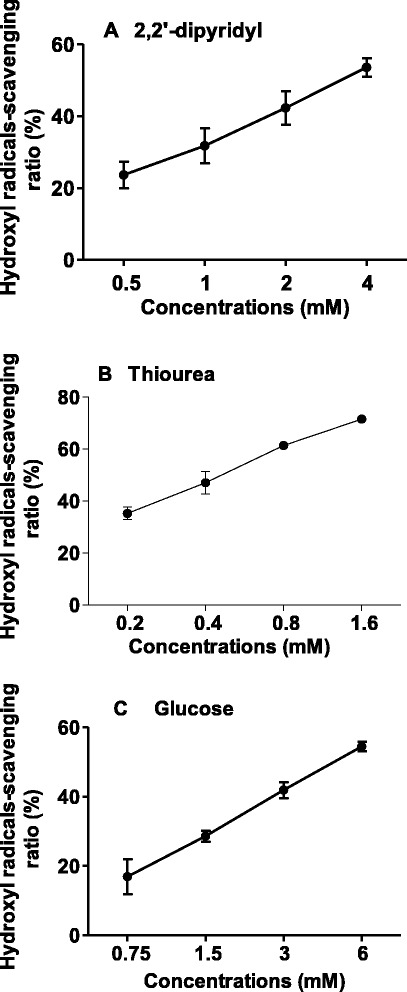
The hydroxyl radicals-scavenging activity of controls. The hydroxyl radicals scavenging activity values represent the percentage of inhibition of deoxyribose degradation. **(A)** 2, 2’-dipyridyl; **(B)** thiourea; **(C)** glucose Data were expressed as means ± S.D. (*n* = 3).

Our previous studies showed that EDF could scavenge hydroxyl radicals *in vitro* (Gao et al. 2010). In order to identify the key residue of EDF to elicit this activity, EDF and its mutants were synthesized by using glycine-scanning strategy, and their hydroxyl-radicals scavenging activity was studied. When the third residue, tryptophan (W), was substituted by glycine (NNGNN), the hydroxyl radicals scavenging activity decreased significantly (Figure 1). In addition, similar results were observed when this residue was substituted by alanine (NNANN) (Figure 2). This activity decrease slightly when each asparagine residue was substituted by glycine. Even all the four asparagine residues of EDF were substituted by alanine, the activity also decreased slightly (Figure 2). Therefore, tryptophan could be the key residue for the hydroxyl radicals-scavenging activity of EDF. In addition, EDF showed more potent hydroxyl radicals-scavenging activity than that of the positive controls, thiourea and 2, 2′-dipyridyl. The potency order is: EDF (IC_50_ ≈ 0.2 mM) > thiourea (IC_50_ ≈ 0.5 mM) > 2, 2′-dipyridyl (IC_50_ ≈ 3.15 mM) (Figure 3) (Mahakunakorn et al. 2004).

The hydroxyl radicals are very active to react with biomolecules. In order to explore how fast EDF and these mutants to scavenge hydroxyl radicals, the reaction rates were also investigated. As shown in Table 1, the reaction rate of EDF was about 26-folds faster than that of glucose, and 3-folds faster than that of the putative hydroxyl radicals-scavenging agent, thiourea. The reaction rate of NNGNN decreased significantly which was similar to that of glucose (Table 1). Interestingly, these results were consistent with another study that tryptophan might be the key residue of EDF to block the MazEF complex and enhance the endoribonucleolytic activity of MazF (Belitsky et al. 2011). These results suggested that the native sequence of EDF (NNWNN) could be important both to the lethal activity and to the hydroxyl radicals-scavenging effects.

**Table 1:** **The kinetics of different compounds to scavenging hydroxyl radicals**

**Compound**	**Second-order rate constants (Ks** ^**a**^ **, M** ^**− 1**^ **s** ^**− 1**^ **)**
EDF	(42.66 ± 7.92) × 10^9^
GNWNN (iEDF)	(20.36 ± 9.41) × 10^9^
NGWNN	(31.47 ± 14.63) × 10^9^
NNGNN	(2.13 ± 1.32) × 10^9^
NNWGN	(14.95 ± 1.96) × 10^9^
NNWNG	(28.87 ± 13.37) × 10^9^
EDF + iEDF	(2.13 ± 0.24) × 10^9^
Thiourea	(13.21 ± 0.99) × 10^9^
2, 2′-dipyridyl	(2.37 ± 0.21) × 10^9^
Glucose	(1.62 ± 0.16) × 10^9^

^a^Ks denotes the rate constant of hydroxyl radical-scavenging reaction determined by scavenging hydroxyl radicals method.

It was reported that the EDF mutant with the first asparagine residue substituted by glycine (GNWNN, iEDF) could inhibit the lethal activity of EDF (Kolodkin-Gal et al. 2007). We discovered that although iEDF could effectively scavenge hydroxyl radicals (Figure 1), it could also remarkably inhibit the hydroxyl radicals-scavenging kinetic process of EDF (Table 1).

### EDF could protect *E. coli* against hydroxyl radicals triggered by bactericidal antibiotics, but not by DNA damage antibiotics

**Figure 4:**
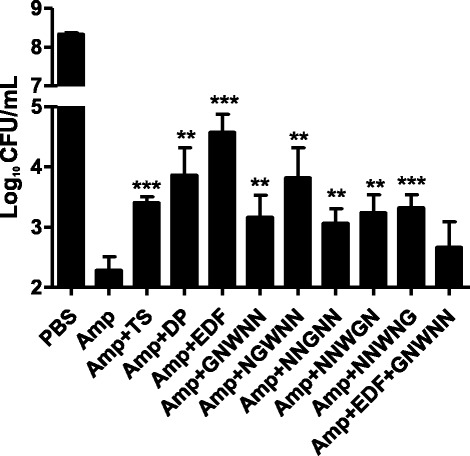
Effects of EDF and its mutants on the killing activity of bactericidal antibiotic, ampicillin, in *E. coli*. The concentration of EDF and its mutants is 0.1 μg/mL. 2, 2′-dipyridyl (DP, 0.5 mM) and thiourea (TS, 150 mM) were used as positive controls. Peptides and positive controls were added along with 15 μg/mL ampicillin (Amp), and incubated at 37°C for 4 hours. CFU/mL was counted and calculated. Data were presented as means ± S.D. (*n* = 4). **p < 0.01, and ***p < 0.001 were presented as compared to the ampicillin group.

Bactericidal but not bacteriostatic antibiotics could promote aerobic biological systems to produce hydroxyl radicals, which ultimately lead to cell death (Kohanski et al. 2007). We investigated the effects of EDF on the bactericidal antibiotic, ampicillin, which could make bacteria produce hydroxyl radicals and finally lead to cell death (Walsh 2003; Kolodkin-Gal and Engelberg-Kulka 2008). As shown in Figure 4, EDF and the positive controls, thiourea and an iron chelator 2, 2′-dipyridyl, could significantly protect *E. coli* from death triggered by ampicillin. When the third residue of EDF was substituted by glycine, the peptide NNGNN exhibited weaker protective effects than that of EDF.

**Figure 5:**
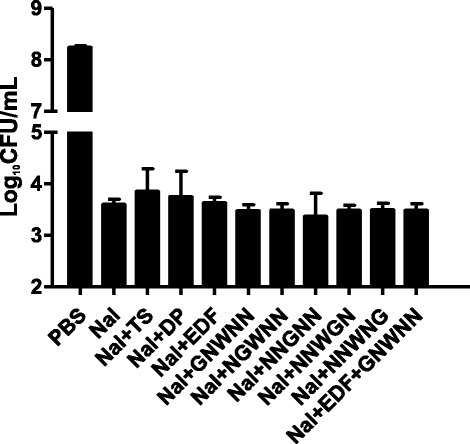
Effects of EDF and its mutants on the killing effects of DNA-damage agent, nalidixic acid, in *E. coli*. The final concentration of nalidixic acid (Nal) was 1 mg/mL. The incubation condition was at 37°C for 3 hours. Other methods were similar to the description in Figure 4. Data were presented as means ± S.D. (*n* = 4).

The other kind of bactericidal antibiotic is DNA-damaging antibiotic which could also trigger ROS-formation, such as nalidixic acid (Kolodkin-Gal et al. 2008). Previous studies demonstrated that nalidixic acid had specific inhibitory effects on the DNA synthesis and led to the cell death of *E. coli* (Goss et al. 1964), but nalidixic acid-mediated cell death could act in an ROS-independent manner (Kolodkin-Gal et al. 2008; Han et al. 2011). As shown in Figure 5, our results showed that EDF and its mutants could not rescue the nalidixic acid-mediated lethal effects on *E. coli*. Although hydroxyl radicals could be induced by nalidixic acid (Kolodkin-Gal et al. 2008), neither the hydroxyl radicals quencher thiourea nor the iron chelator 2, 2′-dipyridyl could neutralize the cell death induced by nalidixic acid.

As expected, the EDF inhibitor iEDF (0.1 μg/mL) could partially inhibit the protective effects of EDF (Figure 4). Although both EDF and iEDF have the ability to scavenge hydroxyl radicals *in vitro* (Figure 1), iEDF could inhibit the reaction rate of EDF *in vitro* (Table 1) which might reduce the efficiency of EDF on scavenging hydroxyl radicals in *E. coli* (Figure 5).

Based on these results, EDF could scavenge hydroxyl radicals *in vitro* and protect *E. coli* against hydroxyl radicals induced by bactericidal agents which could kill cells in an ROS-dependent pathway. The third residue tryptophan (W) could be the key residue of EDF to elicit these activities.

### EDF could not rescue the lethal effects of bacteriostatic antibiotics on *E. coli*

**Figure 6:**
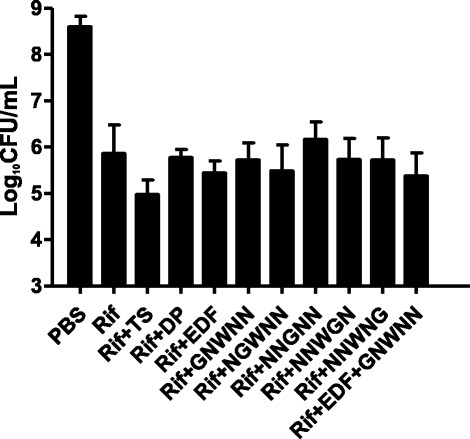
Effects of EDF and its mutants on the inhibition effects of bacteriostatic agent, rifampicin, in *E. coli*. The final concentration of rifampicin (Rif) was 40 μg/mL. The incubation was at 37°C for 4 hours. Other methods were similar to the description in Figure 4. Data were presented as means ± S.D. (*n* = 4).

Kohanski et al. proved that the bacteriostatic antibiotics could not produce hydroxyl radicals in halting bacterial rapid growth (Kohanski et al. 2007). So we investigated whether EDF could protect *E. coli* treated by the bacteriostatic antibiotics, such as rifampicin (Davies and Webb 1998). As shown in Figure 6, EDF and its mutants could not affect the lethal activity of rifampicin (40 μg/mL).

### Effects of EDF on the hydroxyl radicals produced in *E. coli* detected by flow cytometer

**Figure 7:**
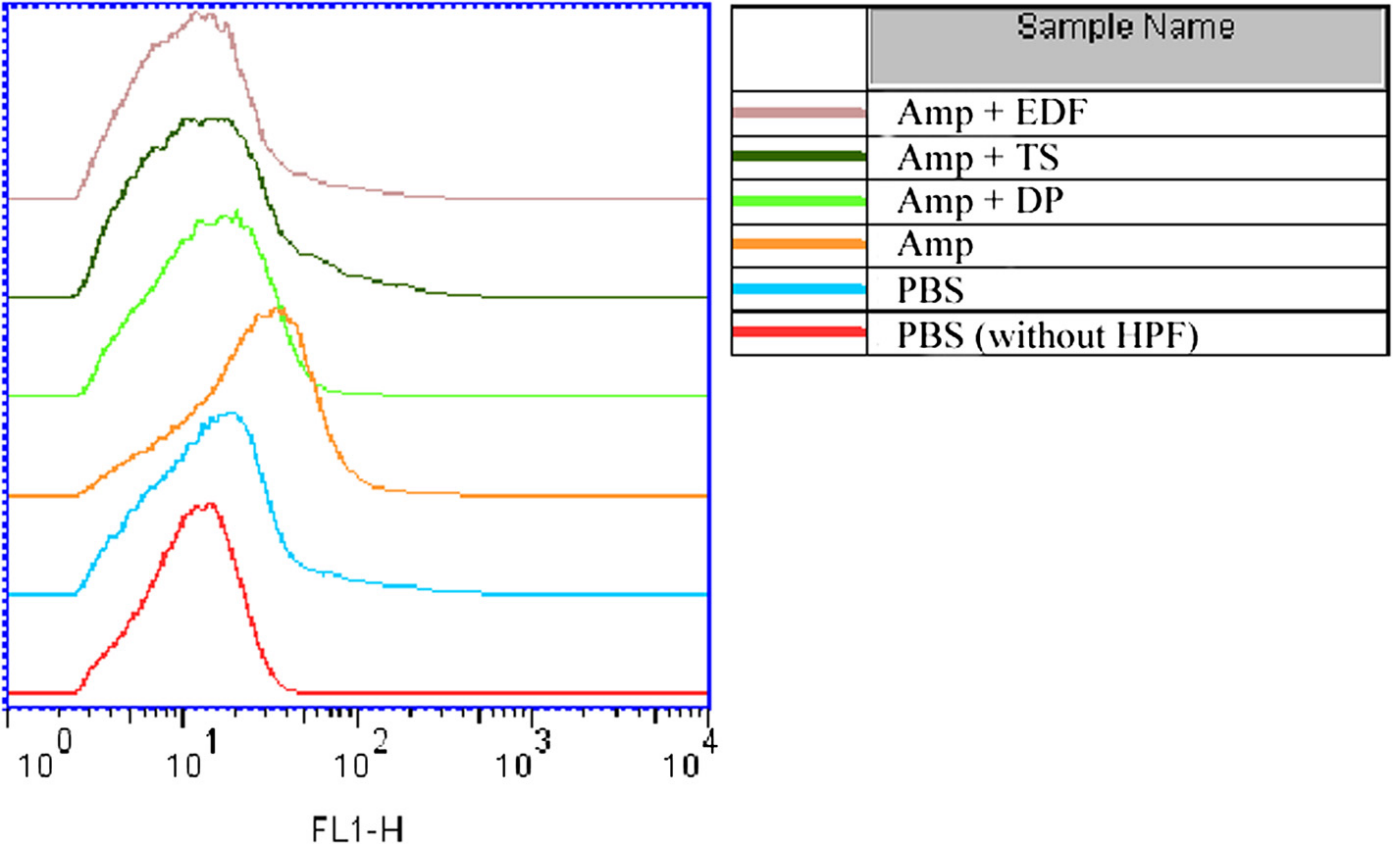
Detection of the hydroxyl radicals produced in *E. coli* treated by EDF under ampicillin stress. The method used was similar to that described in Figure 4. The final concentrations of ampicillin (Amp) and HPF were 15 μg/mL and 5 μg/mL, respectively. After the incubation at 37°C for 4 hours without shaking, cells were collected and fluorescence density was analyzed on a flow cytometer.

Having found that EDF could scavenge hydroxyl radicals *in vitro* (Figure 1) and protect *E. coli* against bactericidal antibiotics-mediated killing (Figure 4), we further investigated whether EDF could scavenge hydroxyl radicals induced by bactericidal antibiotics in *E. coli*. By using a probe 3′-(*p*-hydroxyphenyl) fluorescein (HPF), hydroxyl radicals can be detected by flow cytometer. As shown in Figure 7, high level of hydroxyl radicals triggered by ampicillin was detected by using the HPF probe. Just like the positive controls thiourea and 2, 2′-dipyridyl, EDF could also scavenge hydroxyl radicals produced in *E. coli*.

## Discussion

EDF could act as a quorum sensing factor to help the bacteria to monitor the presence of each other and to modulate post-transcription expression of genes in response to population density (Kolodkin-Gal et al. 2007). EDF could also participate in *mazEF*-mediated PCD in *E. coli* (Aizenman et al. 1996) by inducing the endoribonucleolytic activity of MazF (Belitsky et al. 2011). Once activated by antibiotics, MazF could cleave mRNA at ACA sites to inhibit protein synthesis, including ROS detoxifying enzymes, resulting most of the bacterial population to undergo bacterial PCD (Engelberg-Kulka and Glaser 1999; Zhang et al. 2003; Engelberg-Kulka et al. 2004, 2006; Kolodkin-Gal et al. 2007).

Recently, Kohanski et al. reported a common mechanism that ROS could mediate the bacterial cell death induced by bactericidal agents (Kohanski et al. 2007). EDF and *mazEF* module could determine the mode of the action of some antibiotics (Kolodkin-Gal et al. 2008). To the bactericidal antibiotics of inhibiting transcription and/or translation, *mazEF*-triggered PCD acts in an ROS-dependent manner. In contrast, to the bacteriostatic antibiotics, *mazEF*-mediated cell death acts in an ROS-independent manner. In this case, EDF might only act as a quorum-sensing factor and play a significant role in killing bacteria in logarithmic phase (Aizenman et al. 1996).

We firstly reported that EDF could scavenge hydroxyl radicals and presented the hypothesis that EDF might have dual effects (“survival” and “death”) in *E. coli* (Gao et al. 2010). In the present study, we discovered that EDF could eliminate hydroxyl radicals and ultimately protect a small subpopulation from the damage of hydroxyl radicals triggered by bactericidal agents, but not by ROS-independent antibiotics including DNA damaging antibiotics. Moreover, the DNA damaging antibiotics could induce apoptotic-like death (ALD), which might be different from *mazEF* pathway but mediated by *recA* and *lexA* (Erental et al. 2012).

**Figure 8:**
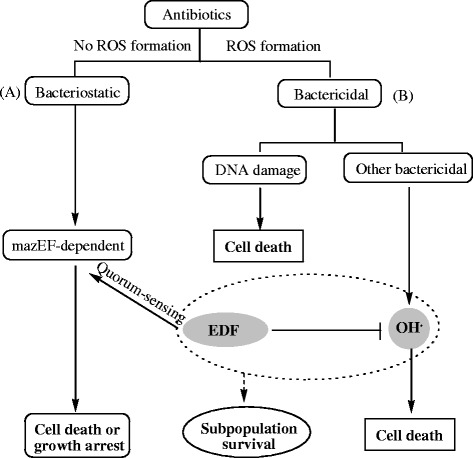
The possible action mode of EDF and antibiotics in *E. coli*.

EDF is the first peptidic quorum-sensing molecule in gram-negative bacteria, and it is derived differently from other signal factors, such as AHLs (Bassler 2002; Taga and Bassler 2003; Henke and Bassler 2004), AI-2 (Bassler 2002; Chen et al. 2002; Federle and Bassler 2003; Taga and Bassler 2003; Xavier and Bassler 2003), 2-heptyl-3-hydroxy-4-quinolone (Pesci et al. 1999; Lazdunski et al. 2004), and indole (Lee et al. 2010). It is derived from the product of the gene *zwf*. We discovered that the natural sequence of EDF might be optimal for its hydroxyl radicals-scavenging activity. As shown in Table 1 and Figure 1, the tryptophan residue plays a vital role in hydroxyl radicals-scavenging activity. It is consistent with the previous report that the tryptophan residue of EDF significantly affected the endoribonucleolytic activities of both MazF and ChpBK *in vitro* (Belitsky et al. 2011). The inhibitor of EDF in quorum-sensing activity, iEDF, could also inhibit the protecting effects of EDF on *E. coli*. All these results indicated that EDF could possibly be a molecule with dual effects (Figure 8).

## Conclusions

In summary, our results revealed that EDF could scavenge hydroxyl radicals *in vitro* and protect *E. coli* from the damage of hydroxyl radicals induced by bactericidal antibiotics. The glycine mutants decreased the hydroxyl radicals-scavenging activity and the protective effects of EDF. The tryptophan residue could be the key residue to the hydroxyl radicals-scavenging and protecting activities of EDF. The “death” and “survival” effects of EDF might relate to the antibiotic resistance in bacteria.

## Abbreviations

EDF: Extracellular death factor
ROS: Reactive oxygen species
NNWNN: Asn-Asn-Trp-Asn-Asn-OH
PCD: Programmed cell death
TS: Thiourea
DP: 2, 2′-dipyridyl
iEDF: EDF inhibitor, GNWNN
HPF: 3′-(p-hydroxyphenyl) fluorescein
ALD: Apoptotic-like death
AHLs: Acyl homoserine lactones
AI-2: Autoinducer-2
CGMCC: China General Microbiological Culture Collection Center
TBARS: Thiobarbituric acid reactive species
LB: Luria-Bertani
CFU: Colony-forming units
Amp: Ampicillin
Nal: Nalidixic acid
Rif: Rifampicin

## References

[CR1] Aizenman E, Engelberg-Kulka H, Glaser G (1996). An Escherichia coli chromosomal “addiction module” regulated by guanosine 3′,5′-bispyrophosphate: a model for programmed bacterial cell death. Proc Natl Acad Sci U S A.

[CR2] Amitai S, Kolodkin-Gal I, Hananya-Meltabashi M, Sacher A, Engelberg-Kulka H (2009). Escherichia coli MazF leads to the simultaneous selective synthesis of both “death proteins” and “survival proteins”. PLoS Genet.

[CR3] Bassler BL (2002). Small talk. Cell-to-cell communication in bacteria. Cell.

[CR4] Belitsky M, Avshalom H, Erental A, Yelin I, Kumar S, London N, Sperber M, Schueler-Furman O, Engelberg-Kulka H (2011). The Escherichia coli extracellular death factor EDF induces the endoribonucleolytic activities of the toxins MazF and ChpBK. Mol Cell.

[CR5] Chen X, Schauder S, Potier N, Van Dorsselaer A, Pelczer I, Bassler BL, Hughson FM (2002). Structural identification of a bacterial quorum-sensing signal containing boron. Nature.

[CR6] Cheng Z, Li Y, Chang W (2003). Kinetic deoxyribose degradation assay and its application in assessing the antioxidant activities of phenolic compounds in a Fenton-type reaction system. Anal Chim Acta.

[CR7] Davies J, Webb V, Richard MK (1998). 8 Antibiotic resistance in bacteria. Biomedical Research Reports, vol. 1. Academic Press.

[CR8] Engelberg-Kulka H, Glaser G (1999). Addiction modules and programmed cell death and antideath in bacterial cultures. Annu Rev Microbiol.

[CR9] Engelberg-Kulka H, Sat B, Reches M, Amitai S, Hazan R (2004). Bacterial programmed cell death systems as targets for antibiotics. Trends Microbiol.

[CR10] Engelberg-Kulka H, Amitai S, Kolodkin-Gal I, Hazan R (2006). Bacterial programmed cell death and multicellular behavior in bacteria. PLoS Genet.

[CR11] Erental A, Sharon I, Engelberg-Kulka H (2012). Two programmed cell death systems in Escherichia coli: an apoptotic-like death is inhibited by the mazEF-mediated death pathway. PLoS Biol.

[CR12] Federle MJ, Bassler BL (2003). Interspecies communication in bacteria. J Clin Invest.

[CR13] Gao Y, Chen K, Zhang B, Li X, Chen L, Li Y, Jia X, Lei Y, Yan Z, Kong L, Wang N, Liu W, Qi Y (2010). Antioxidant and free radical-scavenging activity of the extracellular death factor in Escherichia coli. Peptides.

[CR14] Goss WA, Deitz WH, Cook TM (1964). Mechanism of action of nalidixic acid on Escherichia coli. J Bacteriol.

[CR15] Gutteridge JM, Maidt L, Poyer L (1990). Superoxide dismutase and Fenton chemistry. Reaction of ferric-EDTA complex and ferric-bipyridyl complex with hydrogen peroxide without the apparent formation of iron (II). Biochem J.

[CR16] Halliwell B, Gutteridge JMC, Aruoma OI (1987). The deoxyribose method: A simple “test-tube” assay for determination of rate constants for reactions of hydroxyl radicals. Anal Biochem.

[CR17] Han X, Geng J, Zhang L, Lu T (2011). The role of Escherichia coli YrbB in the lethal action of quinolones. J Antimicrob Chemother.

[CR18] Henke JM, Bassler BL (2004). Bacterial social engagements. Trends Cell Biol.

[CR19] Kohanski MA, Dwyer DJ, Hayete B, Lawrence CA, Collins JJ (2007). A common mechanism of cellular death induced by bactericidal antibiotics. Cell.

[CR20] Kolodkin-Gal I, Engelberg-Kulka H (2008). The extracellular death factor: physiological and genetic factors influencing its production and response in Escherichia coli. J Bacteriol.

[CR21] Kolodkin-Gal I, Hazan R, Gaathon A, Carmeli S, Engelberg-Kulka H (2007). A linear pentapeptide is a quorum-sensing factor required for mazEF-mediated cell death in Escherichia coli. Science.

[CR22] Kolodkin-Gal I, Sat B, Keshet A, Engelberg-Kulka H (2008). The communication factor EDF and the toxin-antitoxin module mazEF determine the mode of action of antibiotics. PLoS Biol.

[CR23] Lazdunski AM, Ventre I, Sturgis JN (2004). Regulatory circuits and communication in Gram-negative bacteria. Nat Rev Microbiol.

[CR24] Lee HH, Molla MN, Cantor CR, Collins JJ (2010). Bacterial charity work leads to population-wide resistance. Nature.

[CR25] Mahakunakorn P, Tohda M, Murakami Y, Matsumoto K, Watanabe H (2004). Antioxidant and free radical-scavenging activity of Choto-san and its related constituents. Biol Pharm Bull.

[CR26] Pesci EC, Milbank JB, Pearson JP, McKnight S, Kende AS, Greenberg EP, Iglewski BH (1999). Quinolone signaling in the cell-to-cell communication system of Pseudomonas aeruginosa. Proc Natl Acad Sci U S A.

[CR27] Taga ME, Bassler BL (2003). Chemical communication among bacteria. Proc Natl Acad Sci U S A.

[CR28] Walsh C (2003). Where will new antibiotics come from?. Nat Rev Microbiol.

[CR29] Xavier KB, Bassler BL (2003). LuxS quorum sensing: more than just a numbers game. Curr Opin Microbiol.

[CR30] Zhang Y, Zhang J, Hoeflich KP, Ikura M, Qing G, Inouye M (2003). MazF cleaves cellular mRNAs specifically at ACA to block protein synthesis in Escherichia coli. Mol Cell.

